# *Propionibacterium acnes* Causing Mediastinal Infection following Endobronchial Ultrasound-Guided Transbronchial Needle Aspiration

**DOI:** 10.1155/2019/3920868

**Published:** 2019-04-07

**Authors:** Katherine Janssen, Joseph Keenan, Roy Cho, Erhan Dincer

**Affiliations:** Division of Pulmonary, Critical Care Medicine, University of Minnesota, Minneapolis, MN, USA

## Abstract

Endobronchial ultrasound-guided transbronchial needle aspiration (EBUS-TBNA) is now a standard of care to sample mediastinal lymph nodes and masses with high diagnostic accuracy and low complication rates. However, the procedure has potential complications that might be life-threatening. Here, we present the first case of *Propionobacterium acnes* (*P. acnes*) causing mediastinitis following EBUS-TBNA of a subcarinal lymph node.

## 1. Introduction

EBUS-TBNA has a pivotal role in diagnosing malignant and benign conditions within the chest. Moreover, it is more cost-effective when compared to mediastinoscopy that is considered gold standard. Real-time needle biopsies can be performed from lymph nodes or masses adjacent to the airway or from the lesions within the lung parenchyma. Besides complications related to bronchoscopy and conscious sedation, EBUS procedure may result in complications related to needle puncturing structures in the chest. These complications are rare and listed as infections (bacteremia, mediastinitis, and pericarditis), pneumothorax, and bleeding.

## 2. Case Report

**Figure 1:**
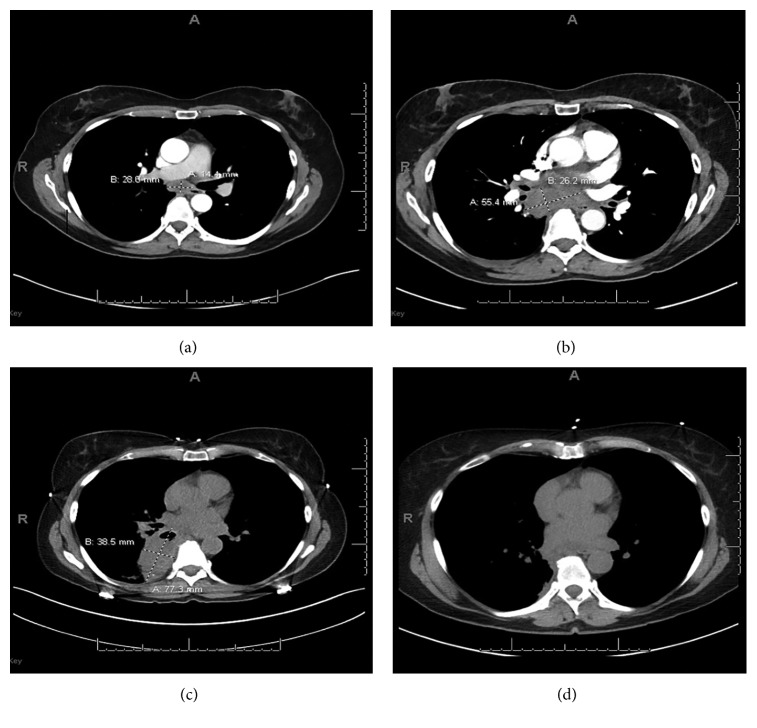
(a) Initial chest CT with newly identified lymphadenopathy. (b) Chest CT 10 days after EBUS with new subcarinal mass. (c) Chest CT 26 days after EBUS with right lower lobe pulmonary abscess. (d) Chest CT following a 6-week course of IV and oral antibiotic therapy (mediastinal and lung windows).

A 57-year-old female with a history of hypothyroidism, hyperlipidemia, and glaucoma presented to the interventional pulmonology (IP) clinic for evaluation of lung nodules found incidentally on chest imaging for evaluation of chronic cough. Her dry cough had persisted for 4 months, and computed-tomography (CT) of the chest demonstrated right lower lobe clusters of noncalcified, solid nodules, largest measuring 10 × 15 mm, with an enlarged subcarinal lymph node (LN) measuring 1.4 × 2.8 cm (Figure 1). She denied fever, chills, anorexia, night sweats, or weight loss. She was a never smoker and had no identifiable environmental or occupational exposures. Her physical examination and initial blood work including complete blood count (CBC) and chemistry were unremarkable. The decision was made to pursue biopsy of the enlarged subcarinal LN to test for old granulomatous disease, in particular, histoplasmosis. Under conscious sedation, endobronchial ultrasound (BF-UC180F bronchoscope) was advanced orally and transbronchial needle biopsy (EBUS-TBNA) of station 7 was performed. A total of 4 biopsies were obtained using a 21G needle (ViziShot Olympus). Rapid on-site evaluation (ROSE) commented on excessive necrosis from each pass. Cultures including bacterial, fungal, and acid-fast bacteria (AFB) were negative. Final cytology was negative for infectious and malignant etiologies.

Ten days later, she presented to the Emergency Room with complaints of a low-grade fever, shortness of breath, and sharp and posterior right-sided chest pain which worsened since the procedure. Her vital signs and physical examination were unremarkable. Initial laboratory work demonstrated a mild leukocytosis with left shift (12.5 × 10^9^/L, 76.9% neutrophils), and CT chest was significant for a large subcarinal mass measuring 5.5 × 2.6 cm causing mass effect on adjacent vessels and esophagus (Figure 1). Following an interdisciplinary discussion with IP, interventional radiology, and thoracic surgery, the decision was to proceed with mediastinoscopy for further evaluation. Intraoperatively, the subcarinal mass had thick fluid inside which grew *Propionibacterium acnes* (*P. acnes*). Fungal cultures, KOH stain, and AFB stain and culture were negative. Final histopathology showed fibroadipose tissue with fibrin, fat necrosis, and chronic inflammation consistent with abscess. There was no evidence of malignancy.

Two weeks later, the patient continued to have a cough and repeat imaging showed a new 7.7 × 3.9 × 8.0 cm irregular, heterogeneous, air-containing mass in the superior medial right lower lobe concerning a lung abscess (Figure 1). The patient was admitted to hospital and begun on IV vancomycin and piperacillin-tazobactam and discharged on IV ampicillin-sulbactam. She was followed up in both IP and infectious diseases clinic 18 days later with significant improvement in symptoms and imaging. She was transitioned to oral amoxicillin-clavulanate for an additional month. Follow-up imaging after completion of antibiotics demonstrated near-resolution of the abscess (Figure 1).

## 3. Discussion

In the early 21st century, endobronchial ultrasound (EBUS) became available and the combined modality of EBUS-TBNA has become first line for the evaluation of mediastinal lymphadenopathy. Since the advent of this technology, EBUS-TBNA has been a safe and well-tolerated procedure with a reported 24 hr complication rate of 1.4%. Although overall complications are rare, development of infectious complications including bacteremia, mediastinitis, pericarditis, and abscess formation following EBUS-TBNA is seldomly reported in the literature [1, 2].

In this current report, we highlight a rare complication following the most common procedure performed by interventional pulmonologists. Our hypothesis is that the pathogen, *P. acnes*, initially discovered through the subcarinal biopsy had disseminated via inoculation of the biopsy needle. Although the benign nature of postbronchoscopy fever is well established, additional symptoms including pleuritic pain and dyspnea should be considered serious and include a thorough evaluation and a multidisciplinary approach as in this case which decidedly contributed to a full recovery. Uniquely, this is the only reported case of *P. acnes* abscess following EBUS-TBNA in an immunocompetent patient.

Currently, there are no accepted recommendations for prophylactic antibiotics with regard to EBUS-TBNA and current management is driven by clinical studies and experience informing expert opinion [3]. Our institution has performed 1500 EBUS-TBNA procedures in the last several years, and a recent study by our group of 210 consecutive EBUS-TBNA procedures had noted 25% granulomatous inflammation on histopathology, 20 of which had necrotizing granulomas [4]. Of these, fungal elements were identified in 30% on Grocott's methenamine silver (GMS) stain and cultures were positive in 15% (3/20) patients (*Mycobacterium tuberculosisn*=1, *Histoplasma capsulatumn*=1, and *Blastomyces dermatitidisn*=1). Although there was no report of post-EBUS infections in our study, there are a total of 18 reported cases of mediastinal infectious complications following EBUS-TBNA with a mean time to infection of 18.1 days [5–9]. Of these, various bacteria have been identified including multiple *Streptococcus* species, *C. albicans*, *P. acnes*, *Klebsiella pneumoniae*, *Gemella morbillorum*, *Prevotella buccae*, *Actinomyces*, *P. aeruginosa*, and diptheroids.

While a mechanism for this complication has not been elucidated, the primary hypothesis is via inoculation of oropharyngeal bacteria into the deep mediastinal tissue during needle puncture. Proposed risk factors include the increasing number of passes in the lymph node tissue, increasing number of samples per lymph node, operator expertise, puncture of a necrotic/cystic lesion (due to decreased bacterial clearance), or contamination of the working channel (due to suctioning in the oropharyngeal cavity above the vocal cords); however, there are no prospective data to support this [6]. Other potential risk factors including bronchoscope sterilization techniques and proper handling of equipment during the procedure should also be considered.

EBUS-TBNA seems to be the optimal sampling modality for mediastinal and hilar lesions; however, serious complications can occur as described in this report. Although infectious complications related to EBUS-TBNA have been accepted to be a rare event, this may actually underscore the inadequacy of adverse event reporting or symptomatology recognition following these procedures. Although there is less certainty to the added benefit of prophylactic antibiotics prior to EBUS-TBNA in all comers, use of antibiotics in select patients where proposed risk factors for procedure-related disseminated infection may improve outcomes. In conclusion, this case demonstrates that post-EBUS infections do occur and may be underrecognized and development of severe symptoms should be identified and managed accordingly.
